# P02-014 - Consequences of Arginine 92 mutations in TNFR1

**DOI:** 10.1186/1546-0096-11-S1-A121

**Published:** 2013-11-08

**Authors:** I Jéru, S Charmion, E Cochet, B Copin, G Le Borgne, P Cathebras, J Gaillat, P Duquesnoy, S Karabina, V Hentgen, S Amselem

**Affiliations:** 1 Service de Génétique, APHP, Hôpital Trousseau, France; 2 Université Pierre et Marie Curie-Paris6, France; 3 UMR_S933, INSERM, Paris, France; 4 Service de Médecine Interne, Centre Hospitalier Universitaire Saint-Etienne, Saint-Etienne, France; 5 Service des Maladies Infectieuses, Centre Hospitalier de la Région d'Annecy, Pringy, France; 6 Centre de Référence des Maladies AutoInflammatoires , Centre Hospitalier de Versailles, Le Chesnay, France

## Introduction

*TNFRSF1A* is involved in a Mendelian autosomal dominant autoinflammatory disorder called *TNFR*-associated periodic syndrome (TRAPS). Most *TNFRSF1A* mutations are missense changes and, apart from those affecting conserved cysteines, their deleterious effect remains often questionable. This is especially true for the frequent R92Q mutation, which might not be responsible for TRAPS per se but represents a susceptibility factor to multifactorial inflammatory disorders.

## Objectives

This study investigates TRAPS pathophysiology in a family exceptional by its size (13 members).

## Methods

*TNFRSF1A* screening was performed by PCR-sequencing. Comparison of the 3-dimensional structure and electrostatic properties of wild-type and mutated TNFR1 proteins was performed by *in silico* homology modeling. TNFR1 expression was assessed by western blotting and ELISA in lysates and supernatants of HEK293T cells transfected with plasmids encoding wild-type and mutated TNFR1.

## Results

A *TNFRSF1A* heterozygous missense mutation, R92W (c.361C>T) perfectly segregated with typical TRAPS manifestations within the family (p<5.10^-4^), and was associated with very high disease penetrance (0.9). Prediction of its impact on protein structure revealed local conformational changes and alterations of electrostatic properties. In addition, R92W leads to abrogation of the receptor shedding, whereas TNFR1-R92Q behaves like the wild-type receptor.

## Conclusion

These data demonstrate the pathogenicity of a mutation affecting arginine 92, a residue whose involvement in inflammatory disorders is deeply debated. Combined with previous data on arginine 92 mutations, this study discloses an unusual situation in which different amino acid substitutions at the same position in the protein are associated with a clinical spectrum bridging Mendelian to multifactorial conditions.

## Disclosure of interest

None declared.

